# A Proteomic Study of Differences in Muscle Quality Between the Longissimus Dorsi and Biceps Femoris Muscles in Junggar Bactrian Camels

**DOI:** 10.3390/biology15131083

**Published:** 2026-07-06

**Authors:** Yongbin Cai, Jintao Gan, Lirong Song, Zhixin Lu, Ye Qin, Wanlu Ren, Jianwen Wang, Xinkui Yao, Jun Meng, Yaqi Zeng

**Affiliations:** 1 College of Animal Science, Xinjiang Agricultural University, Urumqi 830052, China; caiyongbin2025@163.com (Y.C.); ganjintao2022@126.com (J.G.); 18299515075@163.com (L.S.); 18199821647@163.com (Z.L.); 17861279710@163.com (Y.Q.); renwanlu@xjau.edu.cn (W.R.); wjw1262022@126.com (J.W.); yxk61@126.com (X.Y.); junm86@sina.com (J.M.); 2 Xinjiang Key Laboratory of Equine Breeding and Exercise Physiology, Urumqi 830052, China

**Keywords:** bactrian camel meat, different muscles, differentially expressed proteins, association analysis

## Abstract

The longissimus dorsi (LD) and biceps femoris (BF) muscles are important meat-producing regions in camels. Investigating differences in meat quality and proteomic profiles between the LD and BF muscles in Junggar Bactrian camels can provide a molecular basis for regulating camel meat quality and genetic improvement. In this study, 20 healthy adult male Junggar Bactrian camels were selected. Following slaughter, muscle samples were collected from the splenius (SP), triceps brachii (TB), LD, external oblique (EO), gluteus medius (GM), and BF. Meat quality parameters (pH, meat color, shear force, drip loss, and cooking loss) were measured. The LD exhibited the highest meat quality among the six cuts, in contrast to the BF, which showed the lowest. Proteomic analysis of LD and BF from 6 Junggar Bactrian camels was conducted to identify proteins associated with meat quality, yielding 81 differentially expressed proteins (DEPs). Gene Ontology (GO) enrichment analysis highlighted several significantly enriched terms among the DEPs (*p* < 0.05), including calcium-dependent phospholipid binding, zinc ion binding, and metal ion binding. Kyoto Encyclopedia of Genes and Genomes (KEGG) enrichment analysis (*p* < 0.05) further indicated notable enrichment in cytoskeletal organization, 2-oxocarboxylate metabolism, and the citric acid cycle. DEPs associated with meat quality were identified, including tubulin α-chain-like 3 and synaptic function regulator FMR1 isoform X15, which can serve as candidate DEPs for shear force. Protein phosphatase 1 regulatory subunit 14C isoform X1 can serve as a candidate differentially expressed protein for pH. Protein phosphatase 1 regulatory subunit 14C isoform X and anchoring protein repeat domain 1 can serve as candidate DEPs for cooking loss. Membrane-associated protein A4 and membrane-associated protein A7 isoform X1, as well as the transcriptional activator of cytochrome c oxidase 1, can serve as candidate DEPs for color a*. These data may serve as a reference for further studies on how different cuts affect meat quality and for practical efforts to improve camel meat quality.

## Simple Summary

Camel meat is an important source of red meat in arid and semi-arid regions. However, inconsistent meat quality and the absence of a standardized classification system limit its market acceptance. This study compared meat quality across different muscle cuts in Junggar Bactrian camels. Based on these comparisons, two cuts with the greatest quality divergence were selected for proteomic sequencing, bioinformatics, and statistical analysis to identify proteins associated with meat quality. The findings of this study reveal differences in the quality of camel meat across different muscle groups. Proteomic bioinformatics analysis identified several differentially expressed proteins strongly associated with meat quality. These proteins will provide data support for in-depth exploration of camel meat classification and related research.

## 1. Introduction

As a valuable livestock resource, camels outlive other domestic animals under extreme conditions, including high temperatures, severe cold, drought, and food scarcity. Camel meat is distinct nutritionally. Compared to other red meats, its fat and cholesterol content is lower, with a relatively high proportion of polyunsaturated fatty acids (especially omega-3s), essential amino acids, minerals (iron, zinc, selenium), vitamins (E, B vitamins), and bioactive compounds (CoQ10, carnosine, glutathione) [1]. Its superior fatty acid profile, notably abundant cis-9, trans-11 oleic acid, compared to beef, may help reduce cardiovascular disease risk. This makes camel meat a healthier red meat option [2]. Therefore, research on camel meat will provide data to support the improvements of high-quality camel meat. In-depth research into the characteristics of camel meat will provide a molecular basis for the classification of camel meat and lay the groundwork for camel breeding.

Meat quality varies across muscle cuts. A study by Kadim I T et al. on the meat quality of dromedary camels found that six muscle cuts, the infraspinatus, TB, LD, BF, semitendinosus, and semimembranosus, exhibited differences in both nutritional composition and quality indicators [3]. Raiymbek et al. [4] analyzed four muscle cuts from dromedary camels, the LD, BF, semitendinosus, and semimembranosus, and found that the LD had lower protein and moisture content than the other cuts, while its fat and ash content were significantly higher than those of the other muscle cuts. Numerous studies have confirmed that meat quality varies across different cuts of camel meat.

Proteomics is an effective method in meat science. The use of this technology to identify biomarkers for evaluating meat quality has become a breakthrough in the field, as it can uncover the underlying biological pathways and molecular mechanisms behind various meat quality traits [5,6]. Studies have revealed that troponin T, myosin, heat shock protein β-1, creatine kinase, and troponin C possess various anti-stress functions. These proteins are highly correlated with meat quality and can serve as biomarkers for muscle tenderness [7]. The breakdown of myosin heavy chain (MHC) appears to depend on the functional structure of myofibrillar proteins and the extent of cytoskeletal protein degradation; consequently, increased MHC degradation enhances muscle tenderness during the maturation process. Zhang M et al. compared meat samples with contrasting drip loss rates and identified 21 DEPs spanning structural proteins, metabolic enzymes, antioxidant enzymes, and stress response proteins, indicating that these proteins are closely related to muscle water-holding capacity [8]. Poulson J et al. used proteomics to analyze the color stability of the LD and psoas major muscles in cattle. The results revealed that the levels of DEPs in sarcoplasmic proteins were correlated with meat color, with antioxidant and chaperone proteins showing the most significant differences, suggesting they could serve as marker proteins for the color of these two muscle types [9]. Wei Wei et al. used proteomics to identify DEPs potentially associated with skeletal muscle types in pigs [10]. This demonstrates that proteomics has been widely applied in research on livestock meat. Therefore, proteomics represents a valuable approach for examining how different muscle cuts contribute to variation in meat quality, with direct implications for quality improvement.

In this study, meat quality was assessed across six muscle regions, and two regions that showed significant differences were selected for proteomic sequencing. DEPs associated with meat quality were identified through statistical analysis. The findings provide a molecular reference for the classification of camel meat and provide data to support the selection and breeding of camels for meat production.

## 2. Research Methods

This experimental protocol and procedure were approved by the Animal Welfare and Ethics Committee of Xinjiang Agricultural University (Animal protocol number: 2025032). For the meat quality study, 20 healthy, physically similar male Junggar Bactrian camels (aged 4–5 years, weighing 450 ± 50 kg) were used as study subjects. From the slaughtered camel carcasses, the following muscles were sampled: the SP, TB, LD, EO, GM, and BF as research materials. For the proteomics sequencing experiments, the LD and BF muscles were randomly selected from 6 of the 20 camels with the highest meat quality scores identified in the previous study. All camels were purchased from the Mu Xingyuan Breeding Farmers’ Professional Cooperative in Urumqi (Urumqi, Xinjiang, China). The experimental camels were fasted for 24 h and deprived of water for 3 h prior to slaughter, which was performed in strict accordance with the “Operational Procedure of Livestock and Poultry Slaughtering—Cattle” (GB/T 19477-2018) [11]. Meat quality parameters were determined in accordance with the “Determination of Livestock and Poultry Meat Quality” (NY/T 1333-2007) [12]. Approximately 500 g of each muscle was collected for meat quality analysis, and 2 g was stored in liquid nitrogen for proteomic analysis.

### 2.1. Test Equipment and Consumables

Tenderness Tester Model C-LM3B (Beijing Tianxiang Feiyu Technology Co., Ltd., Beijing, China); BS200S Analytical Balance (Sartorius, Göttingen, Germany); pH Meter (Dongguan Wanchuang Electronic Products Co., Ltd., Dongguan, China); 3nh Colorimeter (Guangdong Sanensi Technology Co., Ltd., Guangzhou, China); muscle samples for testing, iRT Kit (Biognosys, Zurich, Switzerland), Bradford Protein Quantification Kit (Beyotime, Shanghai, China), Mass Spectrometry-Grade Trypsin (Promega/V5280, Promega (Beijing) Biotech Co., Ltd., Beijing, China), Triethylammonium Bicarbonate Buffer (TEAB, Sigma/T7408-500ML, Sigma-Aldrich(Shanghai)Trading Co., Ltd., Shanghai, China), LC-MS grade acetonitrile (Thermo Fisher Chemical/A955-4, Thermo Fisher Scientific, Wilmington, DE, USA), LC-MS grade formic acid (Thermo Fisher Scientific/A117-50, Thermo Fisher Scientific, DE, USA), High Abundance Protein Removal Kit (ProteoMiner, Bio-Rad Laboratories (Shanghai) Co., Ltd., Shanghai, China), Low Abundance Protein Enrichment Kit (Bio-Rad/1633007, Bio-Rad Laboratories (Shanghai) Co., Ltd., Shanghai, China), High Select Top 14 Abundant Protein Depletion Mini Spin Columns (Thermo Fisher/A36370, Thermo Fisher Scientific, DE, USA), Large-pore-size filter membrane (Merck/SCNY00020, Merck Investment (China) Co., Ltd., Shanghai, China). Vanquish Neo UHPLC Ultra-High Performance Liquid Chromatography System (Thermo Fisher), Orbitrap Astral Mass Spectrometer (Thermo Fisher), Refrigerated Centrifuge (Scilogex/D3024R, SCILOGEX, LLC, Rocky Hill, CT, USA), freeze dryer (Labogene/Scan Speed 40, LaboGene, Copenhagen, Denmark), electrophoresis unit (Bio-Rad), electrophoresis tank (Bio-Rad), electronic balance (Sartorius/BSA124S, Sartorius Corporate, Germany, Göttingen, Germany), Vortex mixer (Guanghe/HY-6B, Changzhou Jintan Jingda Instrument Manufacturing Co., Ltd., Changzhou, China), microplate reader (Thermo Fisher/MultiskanFC), ice maker (Xueke, Changshu Xueke Electric Co., Ltd., Changshu, China), tissue grinder (Shanghai Jingxin/24-well, Shanghai Jingxin Industrial Development Co., Ltd., Shanghai, China), ultrasonic cell disruptor (Ningbo Xinzhi/JY92-11N, Ningbo Scientz Biotechnology Co., Ltd., Ningbo, China), vacuum filter (Jinteng/GM-0.5A, Tianjin Jinteng Experiment Equipment Co., Ltd., Tianjin, China).

### 2.2. Methods for Assessing Meat Quality

#### 2.2.1. pH Measurement (24 h Post-Slaughter)

After the pH meter was preheated and calibrated, three measurements were taken at evenly spaced points in the center of the meat sample, and the mean pH value was recorded.

#### 2.2.2. Meat Color Detection

After the colorimeter was calibrated with white and black reference plates, three color measurements were taken at evenly spaced points on muscle fibers oriented perpendicular to the surface, and the mean value was recorded.

#### 2.2.3. Determination of Shear Force in Meat

The meat sample was cut into uniform cubes (3 × 1.5 × 1.5 cm^3^) with all visible fascia carefully removed. The cubes were then placed in an 80 °C water bath until the core temperature reached 70 °C (approximately 30 min). Once cooled, the shear force was measured three times using a C-LM3B tenderness tester, and the mean value was recorded.

#### 2.2.4. Cooking Losses

Three 2.5 × 2.5 × 2.5 cm^3^ samples of meat without the outer muscle membrane, each weighing approximately 10–30 g, were taken. Samples were labeled and weighed (m1), then heated in an 80 °C water bath for approximately 30 min until the internal temperature reached 70 °C. Once cooled, surface moisture was removed by blotting with filter paper, and the samples were weighed again (m2). Cooking loss was then calculated using Equation (1).

Cooking loss = (m1 − m2)/m1 × 100(1)

In the formula, the units of mass for m1 and m2 are grams (g).

#### 2.2.5. Drip Loss

After the outer muscle membrane was removed, the meat was cut into three strips measuring 2 × 2 × 4 cm^3^, each weighing approximately 10–30 g. The pre-hanging weight (m1) of each strip was weighed on a balance, and the strips were labeled. Each strip was hung by one end using a hook, stored in a refrigerator at 4 °C for 24 h, and then the post-hanging weight (m2) of each strip was weighed. The results were calculated using Equation (2).

Drip loss = (m1 − m2)/m1 × 100(2)

In the formula, the units of mass for m1 and m2 are grams (g).

### 2.3. Astral DIA Quantitative Proteomics Assay

#### 2.3.1. Total Protein Extraction

Muscle tissues were retrieved from −80 °C storage and pulverized into a fine powder under low-temperature conditions. The powder was rapidly transferred into pre-cooled centrifuge tubes in liquid nitrogen. An appropriate volume of SDT buffer (supplemented with 100 mM NaCl) was added, along with DTT at a final concentration of 1/100 (*v*/*v*), to fully solubilize the samples. After thorough vortex mixing, the lysates were sonicated in an ice-water bath for 5 min to achieve complete cell disruption [13,14]. After sonication, samples were centrifuged at 12,000× *g* for 15 min at 4 °C, and the resulting supernatant was carefully collected. The supernatant was then heated at 95 °C for 8–15 min, briefly cooled on ice for 2 min, and incubated with IAM solution in the dark for 1 h.

Subsequently, four volumes of pre-chilled acetone (−20 °C) were added for protein precipitation, and the mixture was incubated at −20 °C for 30 min. After centrifugation at 12,000× *g* for 15 min at 4 °C, the precipitated protein pellet was collected. The pellet was washed by resuspension in 1 mL of cold acetone (−20 °C), followed by another centrifugation step under the same conditions. Finally, the supernatant was discarded, and the pellet was air-dried. The resulting precipitate was defined as the total protein fraction. An appropriate volume of dissolution buffer (DB buffer) was then added to fully re-dissolve the protein pellet for further analysis.

#### 2.3.2. Protein Detection

Protein concentration was determined using a Bradford assay kit following the manufacturer’s instructions. A bovine serum albumin (BSA) standard curve was prepared with serial dilutions covering a concentration range of 0–0.5 µg/µL. For measurement, different dilutions of both BSA standards and experimental samples were added to a 96-well plate, with each adjusted to a final volume of 20 µL and prepared in triplicate. Then, 180 µL of G250 dye reagent was added to each well immediately. The plate was incubated at room temperature for 5 min, after which absorbance was recorded at 595 nm. A standard curve was generated based on the BSA readings, and protein concentrations of the samples were calculated accordingly. For SDS-PAGE analysis, 20 µg of protein from each sample was loaded onto a 12% polyacrylamide gel. Electrophoresis was carried out at 120 V for 20 min during the stacking phase, followed by 150 V for 50 min for the resolving phase. After separation, gels were stained with Coomassie Brilliant Blue R-250 and subsequently destained until protein bands became clearly distinguishable.

#### 2.3.3. Proteolytic Digestion [15]

Protein samples were first diluted with DB lysis buffer (6 M urea, 100 mM TEAB, pH 8.5) to a final volume of 100 μL. Trypsin and 100 mM TEAB buffer were then added, and the mixture was gently mixed and incubated at 37 °C for 4 h to allow enzymatic digestion. After digestion, formic acid was added to lower the pH to below 3, followed by thorough mixing. The reaction mixture was centrifuged at 12,000× *g* for 5 min at room temperature, and the resulting supernatant was carefully collected. The supernatant was subsequently loaded onto a C18 desalting column. The column was washed three times using a washing solution containing 0.1% formic acid and 3% acetonitrile. Peptides were then eluted using an elution buffer consisting of 0.1% formic acid and 70% acetonitrile. Finally, the eluate was collected and lyophilized for downstream analysis.

#### 2.3.4. DIA Mode LC-MS Analysis

LC-MS/MS analysis was performed using a Vanquish™ Neo UHPLC system coupled with an Orbitrap Astral mass spectrometer. Mobile phase A consisted of 99.9% water with 0.1% formic acid, while mobile phase B consisted of 80% acetonitrile with 0.1% formic acid. Lyophilized peptide samples were reconstituted in 10 μL of mobile phase A, followed by centrifugation at 4 °C and 14,000× *g* for 20 min. A total of 200 ng of the resulting supernatant was injected for LC-MS analysis. Separation was carried out using a Vanquish Neo UHPLC system equipped with a C18 trap column (5 mm × 300 µm, 5 µm; Cat. No. 174500, Thermo Fisher Scientific) and an ES906 PepMap™ Neo UHPLC analytical column (150 µm × 15 cm, 2 µm; Thermo Fisher Scientific). The column oven temperature was maintained at 50 °C. Chromatographic separation was performed under gradient elution conditions as listed in Table 1. Mass spectrometric detection was conducted on an Orbitrap Astral instrument equipped with an Easy-spray electrospray ionization (ESI) source. The spray voltage was set at 2.0 kV, and the ion transfer tube temperature was maintained at 290 °C. Data were acquired in data-independent acquisition (DIA) mode. The MS1 scan range was set to *m*/*z* 380–980. MS/MS spectra were acquired at a resolution of 240,000 (at *m*/*z* 200), with an AGC target of 500%, a precursor isolation window of 2 Th, 300 DIA windows, and a normalized collision energy (NCE) of 25%. For MS2 acquisition, the scan range was set from *m*/*z* 150 to 2000, with a resolution of 80,000 and a maximum injection time of 3 ms. All raw mass spectrometry data were recorded in raw format for subsequent analysis.

#### 2.3.5. Bioinformatics Analysis

Raw DIA data files were processed using DIA-NN software (DIA-NN 1.8.1) against the 3053309_GCF_009834535.1_BCGSAC_Cfer_1.0.fasta protein sequence database. Database search parameters were configured as follows: precursor and fragment mass tolerances were automatically estimated and corrected by the software; carbamidomethylation of cysteine residues was set as a fixed modification; N-terminal methionine excision was considered as a variable modification; and up to two missed cleavages were permitted. To ensure data reliability, DIA-NN post-search filtering was applied. Only peptides with a global Q-value < 0.01 and proteins with a PG.Q-value < 0.01 were retained for further analysis. DEPs were defined based on a threshold of *p* < 0.05 and |log2 fold change| > 1.

Functional annotation of identified proteins was performed using InterProScan software (interproscan-5.22-61.0) to classify protein families and infer associated biological functions [16]. For the DEPs, volcano plot visualization and hierarchical clustering heatmap analysis were conducted to assess overall expression patterns [17]. In addition, Gene Ontology (GO) and Kyoto Encyclopedia of Genes and Genomes (KEGG) enrichment analyses were performed to identify significantly associated biological processes and pathways. Protein–protein interaction (PPI) networks were further constructed using the STRING database (http://STRING.embl.de/) to explore potential functional interactions among DEPs [18].

### 2.4. Statistical Analysis

Meat quality data were analyzed using one-way ANOVA in SPSS 23.0, with results reported as mean ± standard deviation (Mean ± SD). Differences in meat quality among Junggar Bactrian camel meat cuts were assessed, with statistical significance defined as *p* > 0.05 (not significant), *p* < 0.05 (significant), and *p* < 0.01 (highly significant). To explore relationships between meat quality and protein levels, we performed correlation analysis using meat quality phenotypes and DEPs from six Junggar Bactrian camels. Pearson correlation coefficients (r) were calculated for LD and BF proteome and meat quality data. Correlations were considered significant at |r| > 0.75 and *p* < 0.05, and highly significant at |r| > 0.80 and *p* < 0.01 [19]. DEPs were then used to construct a protein–protein interaction network and identify its key nodes.

## 3. Results

The meat quality varies across six muscle cuts of the Junggar Bactrian camel; the results are shown in Table 2.

### 3.1. Shear Force

Shear force values varied among the different muscle groups, ranging from 11.34 to 18.49 kgf. Among these muscles, the SP had the highest shear force, while the LD had the lowest. The shear forces of SP and TB were significantly greater than those of EO, GM, and LD (*p* < 0.01). In addition, SP exhibited a higher shear force value than BF (*p* < 0.05). BF showed significantly higher shear force than EO (*p* < 0.05) and markedly higher values than GM and LD (*p* < 0.01). EO also presented a significantly higher shear force value compared with LD (*p* > 0.05).

### 3.2. pH

pH values differed among the muscle groups, ranging from 5.51 to 5.72. Of these muscles, the SP had the highest pH, while the BF had the lowest. The SP and EO were significantly higher than the TB and BF (*p* < 0.01). The SP exhibited a significantly higher value than LD (*p* < 0.05), while EO showed a significantly higher value compared with GM (*p* < 0.05). LD presented significantly higher values than BF (*p* < 0.01) and TB (*p* < 0.05). No significant differences were observed between LD and either EO or GM (*p* > 0.05). In addition, GM showed a significantly higher value than BF (*p* < 0.05), whereas no significant difference was detected between TB and BF (*p* > 0.05).

### 3.3. Drip Loss Rate

No significant differences in drip loss were observed among the various muscle groups (*p* > 0.05); however, the results show that the drip loss rate was lowest for LD and highest for BF, indicating that LD tends to have higher water-holding capacity than BF.

### 3.4. Cooking Loss Rate

Cooking loss rates differed significantly among the muscle cuts. Among these cuts, TB had the highest cooking loss rate, while LD had the lowest. The cooking loss rate of TB was significantly higher than that of SP, EO, and LD (*p* < 0.01) and significantly higher than that of GM (*p* < 0.05). BF was significantly higher than EO and LD (*p* < 0.01) and higher than SP (*p* < 0.05). GM was significantly higher than EO and LD (*p* < 0.01). SP was significantly higher than LD (*p* < 0.01) and higher than EO (*p* < 0.05). There were no significant differences between TB and BF, between BF and GM, between GM and SP, and between EO and LD (*p* > 0.05).

### 3.5. Meat Color

No significant differences were observed in the L* and b* values among the muscle samples (*p* > 0.05). However, significant differences were found in the a* values. The SP sample had the highest a* value, while the TB sample had the lowest. SP, EO, and LD were significantly higher than BF and TB (*p* < 0.01), and SP was significantly higher than GM (*p* < 0.05). There were no significant differences among SP, LD, and EO (*p* > 0.05). No significant differences were observed between BF and TB (*p* > 0.05). There were no significant differences among LD, EO, and GM (*p* > 0.05).

### 3.6. Protein Identification and Quantification

In proteomics analysis, 48,140 peptides were identified across 12 samples, and 4591 proteins were identified and quantified. Peptide retention times showed low variability, confirming instrument performance stability. The ratio of unique peptides to total peptides was 1:1, confirming the reliability of protein identification. These results indicate that the protein detection data were of adequate quality for subsequent quantitative analysis.

### 3.7. Analysis of DEPs

**Figure 1:**
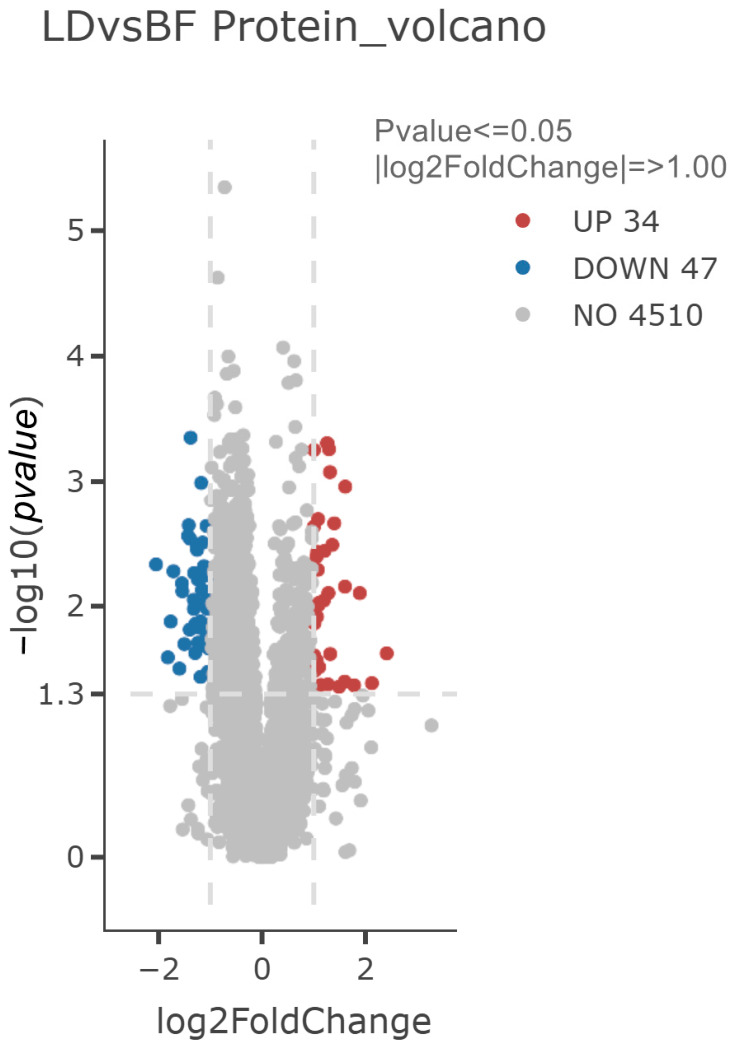
Volcano plot of differentially expressed proteins between LD and BF. The x-axis indicates log2 fold change (|log2FC| > 1), and the y-axis indicates −log10(*p*-value). Red and blue dots represent significantly upregulated and downregulated proteins, respectively (*p* < 0.05).

**Figure 2:**
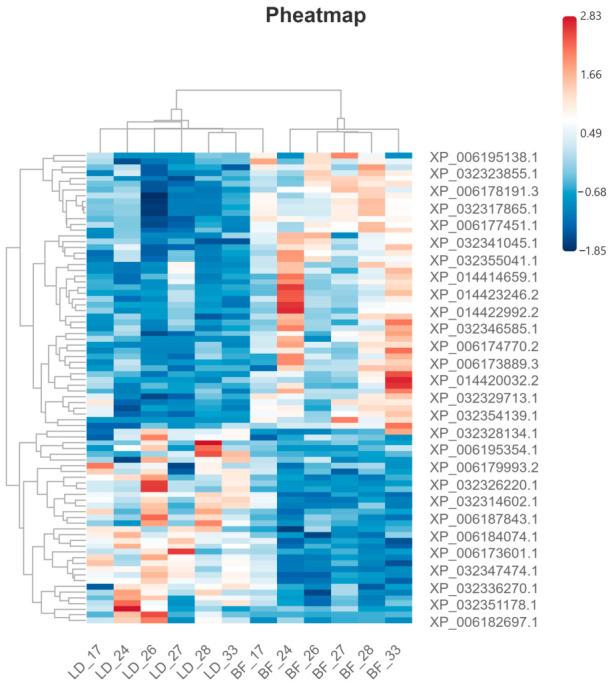
Heatmap of differentially expressed protein clusters. Each column on the x-axis represents a group of samples, and the y-axis shows the relative expression levels of the differentially expressed proteins (log2-transformed). Shorter cluster branches on the left indicate higher similarity among differentially expressed proteins; shorter cluster branches at the top indicate higher similarity among differentially expressed proteins across samples. The more orange the color, the higher the expression level; the more blue, the lower the expression level.

A total of 4591 proteins were identified in both LD and BF, of which 81 exhibited significant changes in expression levels: 34 were significantly upregulated, and 47 were significantly downregulated (Figure 1). Hierarchical clustering analysis revealed distinct expression patterns between LD and BF (Figure 2).

### 3.8. GO Enrichment Analysis of DEPs

**Figure 3:**
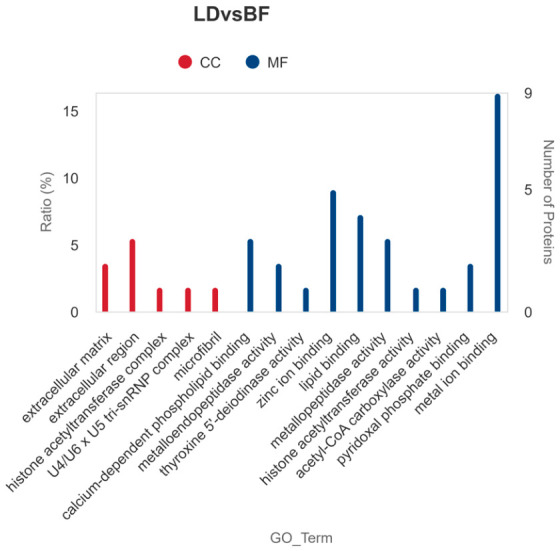
GO Enrichment Bar Chart. Bar chart showing GO enrichment analysis of DEPs. The left-hand axis represents Gene Ratio; the right-hand vertical axis shows the number of annotated differentially expressed proteins; the horizontal axis lists GO Term names; and different colors indicate different functional categories.

The DEPs identified by comparing LD and BF were significantly enriched in 15 terms (Figure 3). The significantly enriched terms were primarily distributed in the “Cellular Component” (CC) category, including extracellular matrix, extracellular region, and histone acetyltransferase complex; and in the “Molecular Function” (MF) category, including metal ion binding, zinc ion binding, and lipid binding.

### 3.9. KEGG Enrichment Analysis of DEPs

**Figure 4:**
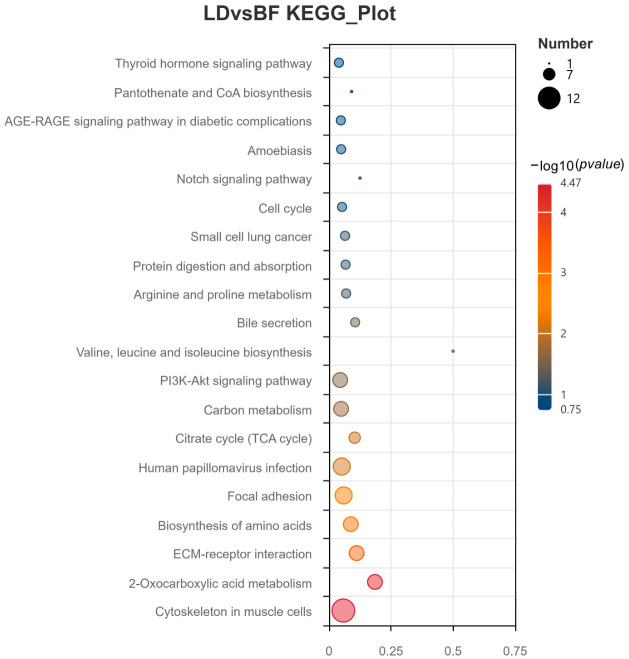
KEGG Enrichment Bubble Chart. The x-axis shows the ratio of the number of DEPs annotated to KEGG pathways to the total number of DEPs; the y-axis shows the KEGG pathways; the size of the circles indicates the number of enriched proteins; and the colors indicate significance.

KEGG pathway enrichment analysis identified 11 significantly enriched pathways among the DEPs between the LD and BF groups (Figure 4). The major enriched pathways were involved in cytoskeletal organization, focal adhesion, and amino acid biosynthesis in muscle cells.

### 3.10. Domain Enrichment Analysis of DEPs

**Figure 5:**
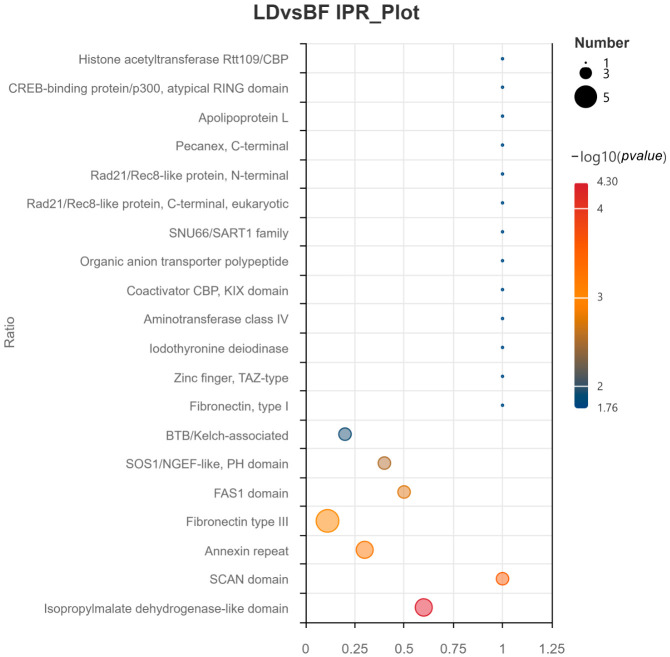
Bubble chart of protein domain enrichment analysis. The x-axis represents the enrichment level (RichFactor = x/y), where higher values indicate stronger enrichment of differentially expressed proteins within a given domain. The y-axis represents protein domain categories.

There was a significant enrichment of 58 protein domains in the DEPs of the LD group compared to the BF group (Figure 5). These included the isopropylmalate dehydrogenase-like domain, the SCAN domain, the annexin repeat, and the fibronectin type III domain, among others.

### 3.11. Analysis of Protein–Protein Interactions (PPI) in DEPs

**Figure 6:**
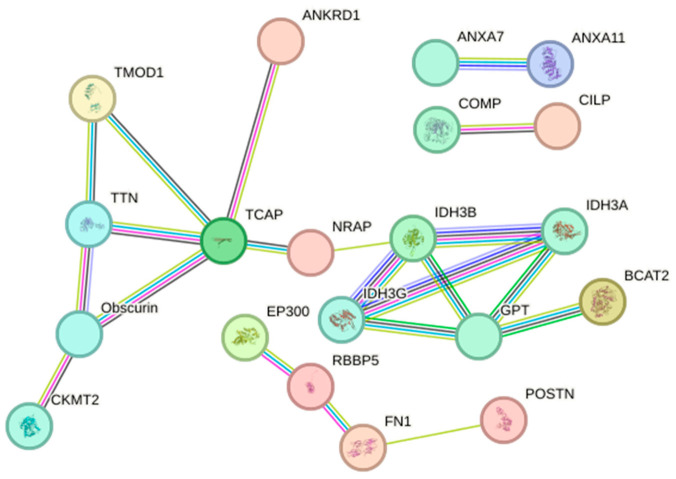
Protein–protein interaction (PPI) network analysis demonstrates the relationships among differentially expressed proteins. The wider the line, the stronger the association with other proteins.

To provide a clearer view of the interactions among these DEPs, a PPI network was constructed based on 81 DEPs (including 34 upregulated and 47 downregulated proteins). As shown in Figure 6, wider and darker lines indicate stronger associations with other proteins. The core of the PPI network constructed from these DEPs includes proteins encoded by genes such as IDH3G, IDH3A, IDH3B, and GPT.

### 3.12. Correlation Between Meat Quality and Differential Proteins

**Figure 7:**
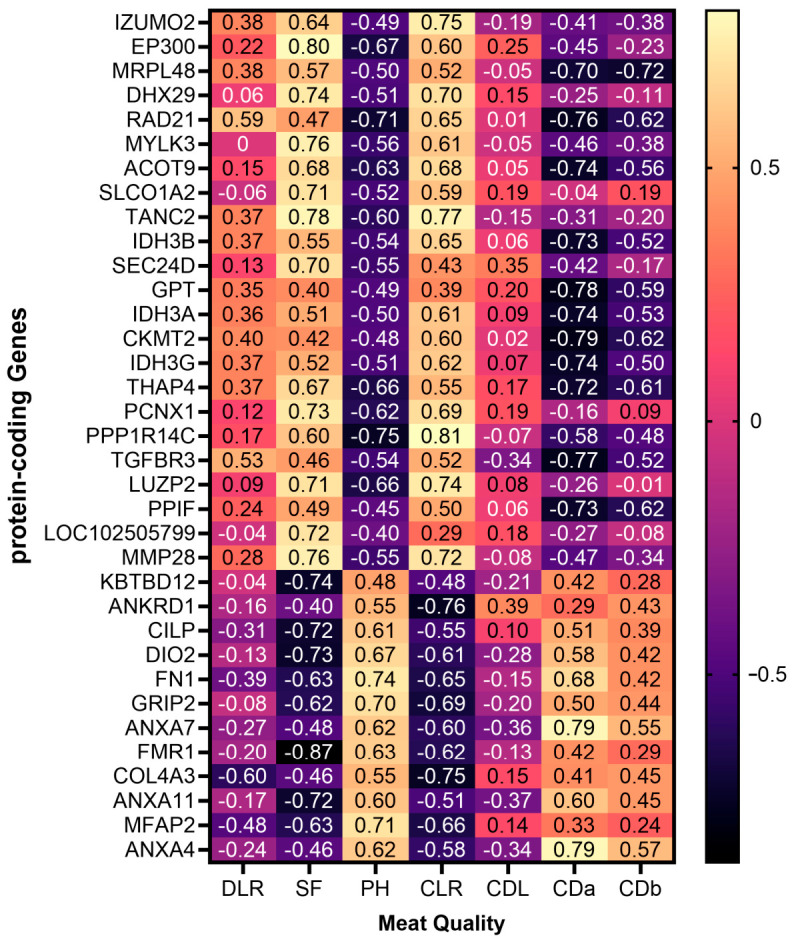
Heatmap showing the correlation between meat quality and differential proteins.For the sake of convenience in plotting, we will abbreviate the drip loss rate as (DLR), shear force as (SF), cooking loss rate as (CLR), color difference L* as (CDL), color difference a* as (CDa), and color difference b* as (CDb).

As shown in Figure 7, a correlation analysis was performed between meat quality and DEPs, and the results with the strongest correlations were plotted. DEPs that showed a significant positive correlation with shear force included myosin light chain kinase 3 subtype X1, protein TANC2 isoform X1, and matrix metalloproteinase 28 isoform X1. Tubulin alpha-chain-like 3 showed a highly significant strong positive correlation, while synaptic function regulator FMR1 isoform X15 showed a highly significant strong negative correlation. Protein phosphatase 1 regulatory subunit 14C isoform X1 showed a significant negative correlation with pH. DEPs significantly positively correlated with cooking loss include IZUMO sperm-oocyte fusion protein 2 subtype X3 and protein TANC2 isoform X1; protein phosphatase 1 regulatory subunit 14C isoform X1 showed a highly significant positive correlation. DEPs significantly negatively correlated with cooking loss include anchoring protein repeat domain 1. Proteins significantly positively correlated with a* colorimetric value include membrin A4 and membrin A7 isoform X1; those significantly negatively correlated include the double-strand break repair protein Rad21 homolog, alanine aminotransferase 1 subtype X1, and mitochondrial creatine kinase S-type, as well as alanine aminotransferase 1 subtype X1. Those showing a highly significant strong negative correlation include the transcriptional activator of cytochrome c oxidase 1.

## 4. Discussion

The meat quality parameters of Junggar Bactrian camels, including shear force, pH, drip loss, cooking loss, and color (L, a, b), were generally consistent with those previously reported for dromedary camels [20,21]. Shear force is one of the core indicators for quantifying meat tenderness, and its value is significantly influenced by genetic factors. Generally, lower shear force values indicate better meat tenderness [22]. According to the study by Destefanis et al. [23], when the shear force value exceeds 5.38 kg·f, beef is considered “tough.” The shear force values for various cuts of Junggar Bactrian camel meat ranged from 11.34 to 18.49 kg·f, indicating that the meat is relatively “tough.” Meat pH directly reflects the acidity or alkalinity of the meat and serves as a key indicator of lactic acid accumulation during muscle glycogenolysis, significantly influencing meat tenderness and shelf life [24]. According to fresh beef grading standards, a pH value between 5.8 and 6.2 indicates optimal meat quality, while a pH below 5.4 is prone to causing PSE (Pale, Soft, Exudative) meat [25]. The muscle pH of various cuts from the Junggar Bactrian camel falls within the normal range. When the a* value of color exceeds 14.5, it typically indicates a higher proportion of oxyhemoglobin in the muscle, excellent redness, and superior meat color quality. The a* values of 14.59–18.64 observed in various cuts of the Junggar Bactrian camel indicate that the meat color quality of these cuts is excellent [26].

Proteomic analysis revealed a significant enrichment of the term “metal ion binding” in the GO enrichment analysis of DEPs between LD and BF. This indicates significant differences between the two muscle types in terms of metal-ion-dependent enzyme activity, protein structure, and signal transduction. These differences are closely related to meat quality attributes such as tenderness, color, and water-holding capacity. Copper ions are essential components of cytochrome c oxidase (a key enzyme in the mitochondrial respiratory chain); differences in their binding capacity directly reflect mitochondrial content and oxidative metabolic capacity, which is central to the distinction between oxidative and fermentative metabolism [27]. Magnesium and zinc ions can bind to myofibrillar proteins, influencing their spatial structure and aggregation state [28,29]. Enrichment results suggest that the structural stability of these proteins may inherently differ between the two muscle types. Calcium ions serve as key messengers for muscle contraction and signal transduction; differences in calcium-binding proteins (such as troponin) directly influence the characteristics of muscle contraction. Additionally, zinc ions participate in the regulation of various signaling pathways, affecting the transformation of muscle fiber types [29].

Proteomic analysis showed that cytoskeleton-related pathways were significantly enriched in the KEGG enrichment analysis of DEPs between LD and BF. The cytoskeleton is essential for muscle fiber formation, structural integrity, contractile function, and the maintenance of fiber type composition in livestock. It is closely associated with muscle fiber characteristics through key structural proteins, including myosin heavy chain (MyHC) and myosin filaments. In skeletal muscle, fiber type composition plays a decisive role in determining multiple meat quality traits, such as color, tenderness, and postmortem pH [30,31]. MyHC is the primary component of myosin molecules, and the expression of its different subtypes (e.g., MyHC I, MyHC IIa, MyHC IIx/d, MyHC IIb) serves as a key molecular marker for distinguishing muscle fiber types [32]. Muscles rich in oxidized Type I and Type IIa muscle fibers are characterized by a bright red color, low shear force, and good tenderness [33]. In contrast, muscles rich in Type IIb muscle fibers exhibit a paler color, a coarser texture, and a slower rate of pH decline [34,35].

The core proteins in the protein–protein interaction network include the isocitrate dehydrogenase [NAD] subunits α, β, and γ, and the mitochondrial subunits (IDH3A, IDH3B, and IDH3G). These three enzymes are the three distinct subunits of the mitochondrial NAD^+^-dependent isocitrate dehydrogenase (IDH3). The function of the IDH3 holoenzyme is to catalyze the oxidative decarboxylation of isocitrate to form α-ketoglutarate, while simultaneously reducing NAD^+^ to NADH. This process is an important rate-limiting step in the TCA cycle [36]. Studies have clearly shown that IDH3 is more abundant in fast-twitch muscle fibers and less abundant in slow-twitch muscle fibers [37]. The higher abundance of this enzyme in BF may be due to metabolic differences between LD and BF, as well as differences in muscle fiber type, which lead to variations in shear stress and pH between the two muscle regions. Sarcomeric proteins (*TTN*, *TCAP*, *ANKRD1*): Tactile protein (*TTN*) forms the core framework of the sarcomere, regulating myofibril assembly and the amplitude of contraction; Troponin C-associated peptide (*TCAP*) contributes to the stability of the troponin complex, enhancing the coordination of slow-twitch muscle contraction; Ankyrin repeat domain protein 1 (*ANKRD1*) localizes to the Z-disks of muscle fibers, where it suppresses the expression of fast-twitch-specific genes while simultaneously enhancing the fatigue resistance of slow-twitch muscle fibers [38]. These three factors work together to maintain the structural stability of slow-twitch muscle fibers in the LD, reduce muscle fiber rupture during contraction, and directly lower shear stress.

DEPs identified in LD compared to BF include deiodinase 2 (*DIO2*), monocarboxylate transporter 4, and transcription activators of cytochrome c oxidase 1, among others. Studies have confirmed that *DIO2* activity in slow-twitch muscle is approximately five times higher than in fast-twitch muscle [39]. As a key protein in the thyroid hormone pathway, *DIO2* catalyzes the conversion of thyroid hormone T4 into active T3, directly activating downstream targets of slow-twitch muscle differentiation, namely myocyte enhancer factor 2C and peroxisome proliferator-activated receptor (MEF2C, PPARδ). MEF2C can activate the promoters of various slow-twitch muscle-specific genes, including the gene encoding the slow myosin heavy chain (*MYH7*); whereas PPARδ regulates genes involved in fatty acid oxidation metabolism, promoting mitochondrial biosynthesis and enhancing oxidative phosphorylation capacity [40]. These proteins confer enhanced aerobic metabolic capacity and superior fatigue resistance in muscle tissue. Additionally, T3 promotes mitochondrial biosynthesis, providing the structural foundation for oxidative metabolism [41], thereby indirectly supporting meat quality phenotypes characterized by high pH and low cooking loss. Monocarboxylate transporter 4 (MCT4) is one of the most direct indicators of glycolytic metabolism in the BF. Studies have shown that MCT4 is a key transporter regulating lactate metabolism in skeletal muscle and is highly expressed in glycolytic fast-twitch muscle fibers, serving as a classic example of muscle adaptation to anaerobic exercise and efficient clearance of metabolic waste [42]. MCT4 is the primary lactate transporter responsible for removing lactate produced by glycolysis from the cell. Its downregulation in the LD (at the protein level) strongly confirms the muscle’s low glycolytic metabolic activity, which is a core manifestation of its aerobic metabolic function [43]. Previous studies have shown that pyruvate kinase (PK) [44], another rate-limiting enzyme in glycolysis, exhibits differences in camel muscle; however, no significant differences in this protein were observed in the present study, which may be due to precise metabolic regulation. Downregulation of MCT4 in the LD and BF proteomes of camels reflects differences in oxidative metabolism between these two sites at the level of key proteins involved in lactate transport, which may account for the low pH in the BF. Transcription factors for cytochrome c oxidase 1 promote the expression of the *COX1* gene. High COX activity accelerates mitochondrial oxygen consumption, exacerbating the hypoxic environment in post-slaughter muscle, which promotes the conversion of myoglobin to metmyoglobin (purple-red). This results in a darker meat color and may lead to a decrease in the a* value [45,46]. This is likely the reason why the transcriptional activator of cytochrome c oxidase 1 shows a significant negative correlation with colorimetric a*. Microtubules, formed by the assembly of tubulin, are one of the three major components of the cytoskeleton. In muscle cells, microtubules play a role in maintaining cell morphology, intracellular transport, and signal transduction. Following slaughter, as the intracellular environment breaks down, the disassembly and degradation of microtubule structures may influence the magnitude of shear stress [6]. This may explain the significant correlation between tubulin and muscle shear stress. PPP1R14C (protein phosphatase 1 regulatory inhibitor subunit 14C) is an endogenous inhibitor of PP1 (protein phosphatase 1); its primary function is to inhibit the dephosphorylation activity of PP1, thereby maintaining the high phosphorylation state of substrate proteins [47]. When PPP1R14C expression is elevated, PP1’s ability to dephosphorylate myofilament proteins is inhibited, resulting in myofilament proteins maintaining higher levels of phosphorylation. Elevated phosphorylation levels of myofilament proteins reduce the kinetic and total energy of the myofilament complex, stabilize the binding conformation between myosin and actin, and promote the formation of ionic bonds, hydrogen bonds, and hydrophobic interactions between them, thereby hindering myofilament dissociation [48]. Impeded myosin-actin dissociation keeps myofilaments in a state of continuous contraction/tight binding, compressing the space between myofibrils and reducing the pore volume available for free water. This is considered the reason why the 14C isoform X1 of protein phosphatase 1 subunit is significantly positively correlated with cooking loss rates [49]. Previous studies have also confirmed that changes in PPP1R14C expression are significantly correlated with cooking loss in meat. In a proteomics study of pork, Yu S et al. clearly demonstrated a significant correlation between PPP1R14C levels and cooking loss [49].

The DEPs identified in this study reflect the differences in meat quality between LD and BF at the molecular level and have preliminarily identified several proteins highly correlated with meat quality. The findings can provide a molecular basis for camel meat classification, and proteins associated with meat quality can be prioritized in future breeding efforts to address the issue of high shear force in camel meat. Some of these proteins are consistent with previous studies, while others differ. These differences reflect variations in camel energy metabolism, particularly in mitochondrial function, and are closely related to camel meat quality. Future research could conduct a combined analysis of meat quality, muscle fiber types, and protein molecules to elucidate, at the molecular level, how proteins influence muscle growth and ultimately lead to differences in meat quality. This experiment has some limitations, and further research is needed to elucidate the specific mechanisms of action of the identified candidate DEPs.

## 5. Conclusions

The study confirmed differences in meat quality among six muscle groups in the Junggar Bactrian camel: the splenius, triceps brachii, longissimus dorsi, external oblique, gluteus medius, and biceps femoris. Among these, the longissimus dorsi exhibited relatively good meat quality, while the biceps femoris exhibited relatively poor meat quality. A total of 81 DEPs were identified across the two muscle regions. These proteins are primarily involved in metal ion binding, as well as pathways related to the muscle cytoskeleton and metabolism. Analysis of DEPs revealed that tubulin α-chain-like 3, synaptic function regulator FMR1 isoform X15, myosin light chain kinase 3 subtype X1, protein TANC2 isoform X1, and matrix metalloproteinase 28 isoform X1 showed strong associations with shear stress and can serve as candidate differential proteins for shear stress. Protein phosphatase 1 regulatory subunit 14C isoform X1 showed a strong correlation with pH and can serve as a candidate differentially expressed protein for pH. IZUMO sperm-oocyte fusion protein 2 subtype X3, protein TANC2 isoform X1, protein phosphatase 1 regulatory subunit 14C isoform X, and anchoring protein repeat domain 1 show a strong correlation with cooking loss rate and can serve as candidate differential proteins for cooking loss rate. Membrane-associated protein A4 and membrane-associated protein A7 isoform X1, double-strand break repair protein Rad21 homolog, alanine aminotransferase 1 subtype X1, creatine kinase type S, alanine aminotransferase 1 subtype X1, and the transcriptional activator of cytochrome c oxidase 1 showed strong correlations with color (a*), and can serve as candidate differential proteins for color (a*). This study explored the mechanisms underlying quality differences in camel meat across different cuts, providing a theoretical basis for further research on improving camel meat quality.

## Figures and Tables

**Table 1:** LC-MS gradient elution settings.

Time	Flow Velocity (μL/min)	Mobile Phase A (%)	Mobile Phase B (%)
0	2.5	96	4
0.2	1.3	96	4
0.3	0.8	92	8
0.5	0.8	92	8
14.2	0.8	77.5	22.5
21.1	0.8	65	35
21.5	2.5	45	55
21.5	2.5	Column Wash	Column Wash
21.9	2.5	1	99
22.6	2.5	1	99
22.6		Stop Run	Stop Run

**Table 2:** Results of Meat Quality Tests.

Meat Quality	SP	TB	LD	EO	GM	BF
Shear force (kgf)	18.49 ± 3.22 ^Aa^	17.93 ± 2.85 ^Aab^	11.34 ± 2.94 ^Cd^	13.63 ± 3.79 ^BCc^	13.06 ± 4.12 ^Ccd^	15.96 ± 2.98 ^ABb^
pH_24 h_	5.72 ± 0.15 ^Aa^	5.54 ± 0.14 ^CDde^	5.63 ± 0.15 ^ABCbc^	5.70 ± 0.13 ^ABab^	5.61 ± 0.10 ^BCDcd^	5.51 ± 0.08 ^De^
Drip loss rate (%)	15.88 ± 2.67 ^a^	16.08 ± 4.00 ^a^	15.75 ± 2.68 ^a^	16.32 ± 3.68 ^a^	16.41 ± 2.64 ^a^	17.02 ± 3.74 ^a^
Cooking loss rate (%)	38.75 ± 3.57 ^BCc^	42.12 ± 1.97 ^Aa^	34.72 ± 4.42 ^Dd^	36.42 ± 3.53 ^CDd^	39.44 ± 3.71 ^ABbc^	41.09 ± 1.87 ^ABab^
Color difference (L*)	28.20 ± 3.84 ^a^	30.16 ± 2.73 ^a^	30.62 ± 3.47 ^a^	29.71 ± 3.23 ^a^	28.28 ± 2.63 ^a^	28.73 ± 2.64 ^a^
Color difference (a*)	18.64 ± 2.35 ^Aa^	14.59 ± 2.40 ^B^	17.63 ± 2.73 ^Aab^	17.73 ± 1.92 ^Aab^	16.92 ± 2.05 ^Ab^	14.70 ± 2.10 ^B^
Color difference (b*)	4.05 ± 1.44 ^a^	3.82 ± 1.16 ^a^	4.76 ± 1.41 ^a^	4.20 ± 1.32 ^a^	4.01 ± 1.30 ^a^	3.53 ± 1.00 ^a^

Note: Different uppercase letters indicate highly significant differences among groups (*p* < 0.01), whereas different lowercase letters represent significant differences (*p* < 0.05). Groups sharing the same letters or without letter annotations show no significant differences (*p* > 0.05).

## Data Availability

Raw reads of proteomic sequencing of camel skeletal muscle are available at CNCB. OMIX submission information: OMIX014968. https://ngdc.cncb.ac.cn/omix/OMIX014968 (accessed on 2 July 2026).

## Abbreviations

DEPs	Differentially expressed proteins
SP	Splenius
TB	Triceps brachii
LD	Longissimus dorsi
EO	External Oblique
GM	Gluteus Medius
BF	Biceps femoris
GO	Gene ontology
KEGG	Kyoto encyclopedia of genes and genomes
PPI	Protein–protein interaction
SF	Shear force
DLR	Drip loss rate
CLR	Cooking loss rate
CDL	Color difference (L*)
CDa	Color difference (a*)
CDb	Color difference (b*)
